# Toxin-Antitoxin Systems - A New Player in Morphological Transformation of Antibiotic-Exposed *Helicobacter pylori*?

**DOI:** 10.3389/fcimb.2021.670677

**Published:** 2021-04-26

**Authors:** Paweł Krzyżek

**Affiliations:** Department of Microbiology, Faculty of Medicine, Wroclaw Medical University, Wroclaw, Poland

**Keywords:** *Helicobacter pylori*, toxin-antitoxin system, coccoid forms, morphological transformation, stress response

## Introduction

The recently published original article by Mortaji et al. (2020) characterized for the first time the function of a type I toxin-antitoxin (TA) system in the gastric pathogen *Helicobacter pylori*. It was found that the high expression of an AapA1 toxin, which is part of this system, causes a drastic decrease in the amount of culturable *H. pylori* cells and their transformation from a spiral to a coccoid morphotype. It was also established that AapA1 is a hydrophobic peptide disrupting cell division and that oxidative stress is an inducer of the toxin expression.

The development of genomics and bioinformatics in recent years has contributed to the discovery of a high frequency of TA systems in microorganisms, which was a strong stimulus for the intensification of research on their structure and function (Lee and Lee, 2016; Yang and Walsh, 2017). Prokaryotic TA modules are genetic elements that encode information about a toxin involved in inhibiting growth of the bacterial producer and an antitoxin that counteracts the activity of the former. Toxins belonging to TA systems restrict microbial replication by targeting key processes for cell physiology, including replication, transcription, translation and/or cell wall synthesis (Harms et al., 2018). Attention is being paid increasingly to the participation of these systems in suppressing the microbial multiplication and the stimulatory effect on adaptation to stressful conditions, i.e., nutritional starvation, exposure to antimicrobial substances or immune system cells’ attack (Lee and Lee, 2016; Yang and Walsh, 2017).

To date, five type II TA systems of *H. pylori* have been identified. These include chromosomally encoded HP0892-HP0893 (Han et al., 2013), HP0894-HP0895 (Han et al., 2011), HP0315-HP0316 (Kwon et al., 2012), and HP0967-HP0968 (Cárdenas-Mondragón et al., 2016), and the newly identified TfiT-TfiA (Boampong et al., 2020), which is encoded on mobile genetic fragments. The expression of toxins belonging to the above modules arrest the growth of bacterial producers and cause the reduction of their number (expressed in CFU/mL). Similar observations were made in 2017 by Arnion et al. (2017), who first identified the existence of the type I TA system in *H. pylori* (called AapA1-IsoA1), and noted that the expression of the toxin significantly decreases the amount of culturable *H. pylori* cells. At this point it is worth mentioning that Mortaji et al. (2020) deepened the knowledge related to the above phenomenon. They proved in their next original article that this decline was caused by a reduction in the culturability (observed as the optical density of the culture) but not the viability of *H. pylori* (preserved cell membrane integrity and a stable ATP level), and was accompanied by the transition of morphology from spiral to coccoidal (Mortaji et al., 2020). This observation is very valuable from the scientific point of view and confirms the postulates presented by our research group, pointing to difficulties in the correct interpretation of the *H. pylori* viability (understood as the sum of various cell parameters suggesting its physiological activity) and frequent mistakes made by scientists taking the culturability (detected by culture optical density or CFU/mL) as the only determinant of the viability of this pathogen (Krzyżek and Grande, 2020).

An additional valuable cognitive element shown by Mortaji et al. (2020) was a proof that oxidative stress was an inducer of the *aapA1* expression in *H. pylori*, and thus a trigger for the spiral-to-coccoid transition. Exposure to high concentrations of oxygen, understood here as oxidative stress, is a well-known stress factor for *H. pylori* determining its intensive transformation into spherical forms (Chuang et al., 2005; Zeng et al., 2008). Thus, Mortaji et al. (2020) neatly revealed a possible molecular mechanism governing this process. In regard to this, it is also worth paying attention to the results presented by many research teams that have shown that bactericidal antibiotics, unlike bacteriostatic ones, stimulate the formation of oxygen free radicals and oxidative stress in bacterial cells, regardless of their target site (Kohanski et al., 2007; Brynildsen et al., 2013; Dwyer et al., 2014; Belenky et al., 2015; Lobritz et al., 2015; Li et al., 2017). According to Lobritz et al. (2015), this effect was particularly visible with the use of antibiotics acting on the microbial cell wall and DNA, but neither translation nor transcription. The above information, in conjunction with the results provided by Mortaji et al. (2020), seem to be extremely interesting, as they may explain why bactericidal antibiotics (amoxicillin, levofloxacin or metronidazole) induce morphological transformation into spherical forms in *H. pylori* significantly faster than bacteriostatic antibiotics (Sörberg et al., 1997; Sörberg et al., 1998; Akada et al., 1999; Faghri et al., 2014; Krzyżek et al., 2019a; Krzyżek et al., 2019b). Still, it should be remembered that the process of cell death and/or formation of coccoids by *H. pylori* during the exposure to bactericidal antibiotics may depend on many factors simultaneously or be independent of oxidative stress.

**Figure 1:**
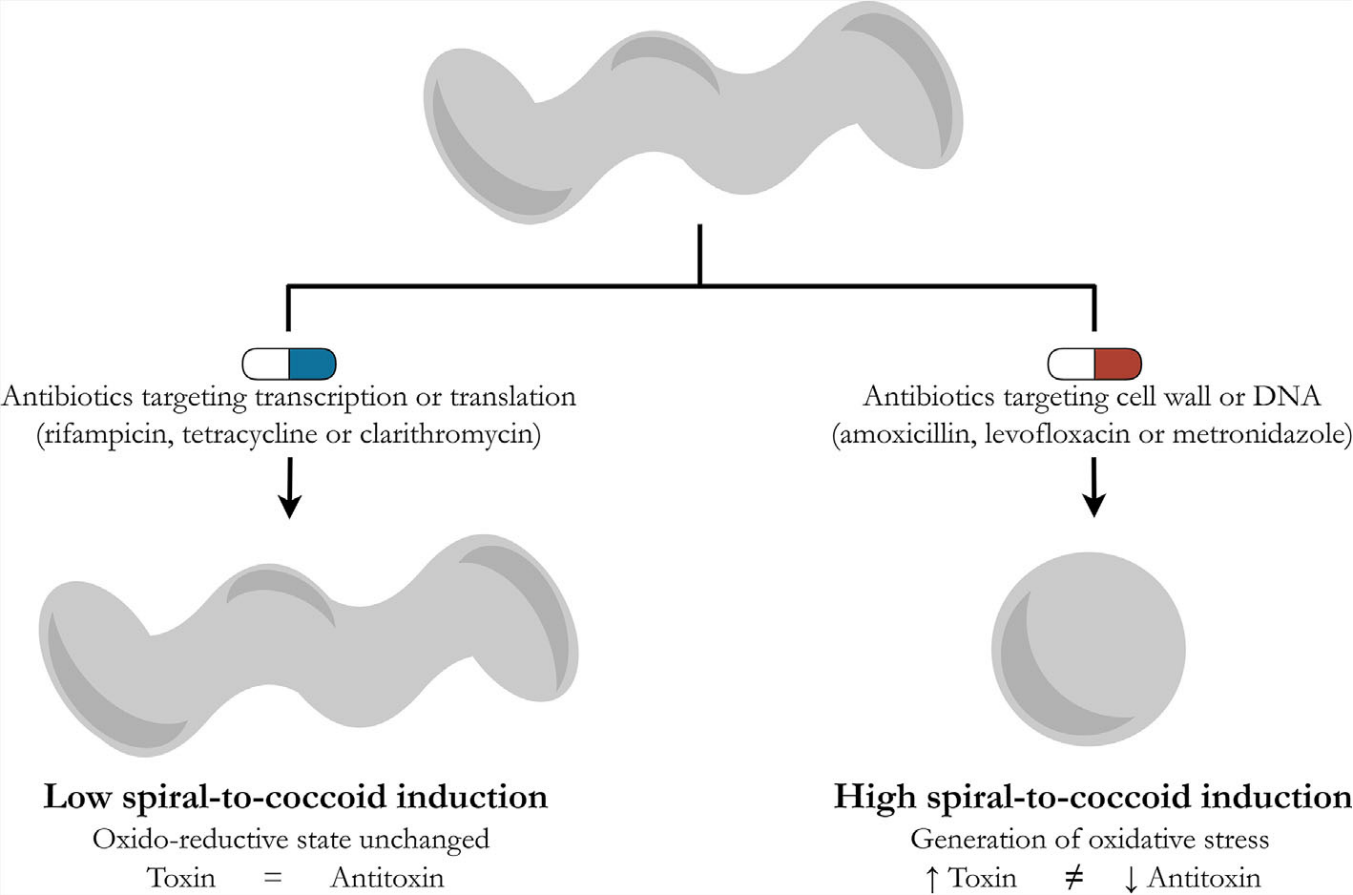
Schematic drawing presenting a hypothetical model describing differences in the potential of antibiotics to generate the spiral-to-coccoid transformation in *H. pylori*. In the routine therapy of *H. pylori*, the following antibiotics are used: rifampicin (transcription), tetracycline and clarithromycin (translation), metronidazole and levofloxacin (the DNA structure or replication), and amoxicillin (the cell wall) (Jones et al., 2008; Francesco, 2011; Nishizawa and Suzuki, 2014). Based on reports showing the ability of bactericidal antibiotics to stimulate oxidative stress in microbial cells (Kohanski et al., 2007; Brynildsen et al., 2013; Dwyer et al., 2014; Belenky et al., 2015; Lobritz et al., 2015; Li et al., 2017) and the results of Mortaji et al. (2020), demonstrating the oxidative stress-dependent induction of the toxin-antitoxin system in *H. pylori*, a hypothetical model integrating the above observations has been proposed. Antibiotics acting on transcription and translation (rifampicin, tetracycline or clarithromycin) have a marginal effect on the oxido-reductive state of bacterial cells and therefore do not significantly affect the toxin-antitoxin balance. The opposite situation is suggested for antibiotics targeting the cell wall or DNA (amoxicillin, levofloxacin or metronidazole), all of which stimulate the accumulation of reactive oxygen species in bacterial cells and the oxidative stress-related disturbance of the toxin-antitoxin balance in favor of the former. The increased production of this toxin is accompanied by the conversion of *H. pylori* into spherical forms.

In the original article by Mortaji et al. (2020), *H. pylori* was exposed to one of two antibiotics: rifampicin or tetracycline targeting transcription or translation, respectively. The authors did not observe any significant increase in the *aapA1* expression in rifampicin- or tetracycline-treated cells, concluding that exposure of *H. pylori* to antibiotics did not affect the expression of this toxin. In the light of the above presented deduction, however, it seems that divergent results may arise for other antibiotics used in the therapy of *H. pylori*, especially those with a strong bactericidal activity, e.g., amoxicillin, levofloxacin or metronidazole. Extending research to include these antibiotics would allow it to be established whether the hypothesis presented by an author of this commentary about the inducing effect of bactericidal antibiotics and their oxidative stress-dependent generation of morphological transition into spherical forms by *H. pylori* is correct (**Figure 1**).

Finally, it is worth noting that the results presented by Mortaji et al. (2020) may have clinically significant implications, especially in the context of the eradication of difficult-to-treat, recurrent *H. pylori* infections. Recently, Morales-Espinosa et al. (2020) showed that the expression of HP0315, one of the components of the type II TA systems, is expressed significantly higher in intracellular *H. pylori* subpopulations and that the expression of this gene was accompanied by the formation of coccoid forms by these bacteria. Therefore, it seems very interesting to determine whether this type of relationship can also be demonstrated for other TA modules, including AapA1-IsoA1, and whether lowering the expression of the toxin or increasing the expression of the antitoxin would positively influence the frequency of *H. pylori* eradication.

## Author Contributions

The author confirms being the sole contributor of this work and has approved it for publication.

## Funding

The study was supported by the Wroclaw Medical University grant No: SUB.A130.21.031. The funder had no role in the preparation of the manuscript.

## Conflict of Interest

The author declares that the research was conducted in the absence of any commercial or financial relationships that could be construed as a potential conflict of interest.
